# Characterization of Nanovesicles Isolated from Olive Vegetation Water

**DOI:** 10.3390/foods13060835

**Published:** 2024-03-08

**Authors:** Sandra Buratta, Raffaella Latella, Elisabetta Chiaradia, Anna Maria Salzano, Brunella Tancini, Roberto Maria Pellegrino, Lorena Urbanelli, Giada Cerrotti, Eleonora Calzoni, Husam B. R. Alabed, Sabrina De Pascale, Luana Lugini, Cristina Federici, Andrea Scaloni, Carla Emiliani

**Affiliations:** 1 Department of Chemistry, Biology and Biotechnology, University of Perugia, 06100 Perugia, Italy; raffaella.latella@studenti.unipg.it (R.L.); brunella.tancini@unipg.it (B.T.); roberto.pellegrino@unipg.it (R.M.P.); lorena.urbanelli@unipg.it (L.U.); giada.cerrotti@studenti.unipg.it (G.C.); eleonora.calzoni@unipg.it (E.C.); husambr.alabed@studenti.unipg.it (H.B.R.A.); carla.emiliani@unipg.it (C.E.); 2 Department of Veterinary Medicine, University of Perugia, 06100 Perugia, Italy; elisabetta.chiaradia@unipg.it; 3 Proteomics, Metabolomics and Mass Spectrometry Laboratory, Institute for the Animal Production System in the Mediterranean Environment (ISPAAM), National Research Council, 80055 Portici, Italy; annamaria.salzano@cnr.it (A.M.S.); sabrina.depascale@cnr.it (S.D.P.); andrea.scaloni@cnr.it (A.S.); 4 Department of Oncology and Molecular Medicine, Preclinical Research and Clinical Trials in Hematology and Oncology Unit, Istituto Superiore di Sanità, 00161 Rome, Italy; luana.lugini@iss.it (L.L.); cristina.federici@iss.it (C.F.); 5 Centro di Eccellenza sui Materiali Innovativi Nanostrutturati (CEMIN), University of Perugia, Via del Giochetto, 06123 Perugia, Italy

**Keywords:** nanovesicles, olive vegetation water, edible plant/fruit-derived extracellular vesicles, lipidomics, metabolomics, proteomics

## Abstract

Edible plant and fruit-derived nanovesicles (NVs) are membrane-enclosed particles with round-shape morphology and signaling functions, which resemble mammalian cell-derived extracellular vesicles. These NVs can transmit cross-kingdom signals as they contain bioactive molecules and exert biological effects on mammalian cells. Their properties and stability in the gastrointestinal tract suggest NVs as a promising nutraceutical tool. In this study, we have demonstrated for the first time the presence of NVs in olive vegetation water (OVW), a waste by-product generated during olive oil production. Biophysical characterization by scanning electron microscopy, cryo-transmission electron microscopy, and nanoparticle tracking analysis revealed the presence in OVW of NVs having size and morphology similar to that of vesicles isolated from edible plants. Integrated lipidomic, metabolomic, and proteomic analyses showed that OVW-NVs carry a set of lipids, metabolites and proteins which have recognized antioxidant and anti-inflammatory activities. The nature of biomolecules identified in OVW-NVs suggests that these vesicles could exert beneficial effects on mammalian cells and could be used in the nutraceutical and food industries. The successful isolation of OVW-NVs and the characterization of their features strengthen the idea that agricultural waste might represent a source of NVs having features similar to NVs isolated from edible plants/fruits.

## 1. Introduction

The isolation and biological characterization of nanovesicles (NVs) from edible vegetables and fruits represent a recent and growing research field in pharmaceutical and nutraceutical areas, as they are vehicle of natural bioactive compounds that can be used to deliver these molecules for medical purposes. NVs are membrane-enclosed nanoparticles, having structure (i.e., cup-shaped morphology and dimensions) and biochemical composition and functions (i.e., intercellular communication) similar to extracellular vesicles derived from mammalian cells and biological fluids [1,2,3]. Depending on their origin, plant- and fruit-derived NVs contain functional macromolecules (i.e., proteins and RNAs) and a set of small metabolites (i.e., lipids, amino acids, sugars, vitamins, carotenoids, and flavonoids) of biochemical relevance [2,4]. The presence of bioactive molecules in NVs from edible plants and fruits has focused the researchers’ attention on the role of these vesicles in intercellular communication, even between different species [5,6]. Recent *in vivo* and *in vitro* studies have demonstrated that plant- and fruit-derived NVs are stable in the gastrointestinal tract and exhibit tissue-specific targeting capabilities [7]. Consistently, bioactive molecules carried by NVs are protected by degradation along the way to recipient cells, where they exert beneficial effects (i.e., antioxidant, anti-inflammatory, and antitumoral activity) [8,9]. Due to their properties, NVs derived from edible plants and fruits nowadays are considered novel promising therapeutic and nutraceutical tools. The characterization of their specific molecular content is crucial to understand their functional properties and to predict their biological effects.

It is well known that the industrial processing of vegetables and fruits generates a huge amount of waste, mainly in the form of wastewater and pomace. These residues still retain a great number of phytochemicals and nutraceuticals exhibiting a wide range of health-promoting effects [10]. Hence, waste from vegetable and fruit processing represents a rich source of natural bioactive compounds for pharmaceutical and cosmetic industries, as well as for the preparation of functional foods. However, the utilization of these compounds is challenging due to their sensitivity to heat, light, pH, and oxygen, which can cause their chemical degradation and inactivation. Furthermore, many of them are degraded in the gastrointestinal tract with consequent reduction in bioavailability and activity [10]. To overcome these problems, recent studies have proposed the application of novel nanotechnological techniques (i.e., micro- and nano-encapsulation) to improve the chemical stability and bioavailability of these bioactive substances and allow their controlled delivery and release [10].

Purification of NVs from agricultural wastes may represent an innovative solution to the above-mentioned problems. In fact, bioactive molecules can reach target cells directly as “phyto-mixture” encapsulated into natural nanoparticles surrounded by membranes, which protect them from degradation. Thus, the direct isolation of NVs from agro-industrial wastes could represent an innovative and eco-friendly technology to obtain bioactive compounds ready for their use and to avoid their recovery through unsafe extraction with organic solvents. In this study, we have isolated NVs from olive vegetation water (OVW), a waste by-product of olive oil production. OVW is commonly used to chemically extract bioactive molecules, such as phenolic compounds, which are further used in the pharmaceutical, nutraceutical, and food industries due to their antioxidant and antimicrobial properties [11]. Here, OVW-NVs have been biophysically characterized through scanning electron microscopy, nanoparticle tracking analysis, and cryo-transmission electron microscopy. Then, OVW-NVs were biochemically characterized by profiling their lipid, metabolite, and protein cargo. Knowledge on biomolecules packaged in OVW-NVs is a crucial step to predicting the biological effects of these vesicles on mammalian cell lines.

This study is part of a promising area of research that aims to develop methods for isolating NVs from agricultural wastes, and to explore their biological properties, in order to use these NVs as tools for nutraceutical applications. Thus, agri-food wastes might become a valuable source of bioactive NVs, reinforcing the concept of waste valorization in the context of the circular economy.

## 2. Materials and Methods

### 2.1. Materials

LC/MS-grade water (H_2_O), acetonitrile (ACN), methanol (MeOH), and isopropanol (IPA) were from Merck (Darmstadt, Germany). Toluene (Tol) was from Carlo Erba (Milan, Italy). Ammonium fluoride, ammonium acetate, methyl-t-butyl ether (MTBE), and chloroform (Chlo) were from Merck Life Science (Hamburg, Germany). Splash Lipidomix from Avanti Polar (Alabaster, AL, USA) was used as internal standard (IS) for lipidomic analysis. It consisted in a mixture with known concentration of the following lipids [nmol/mL]: phosphatidylcholine (PC) 15:0_18:1(d7) [212.6]; phosphatidylethanolamine (PE) 15:0_18:1(d7) [7.0]; phosphatidylserine (PS) 15:0_18:1(d7) [6.6]; phosphatidylglycerol (PG) 15:0_18:1(d7) [40.5]; phosphatidylinositol (PI) 15:0_18:1(d7) [12.1]; phosphatic acid (PA) 15:0_18:1(d7) [10.5]; lyso-phosphatidylcholine (LPC) 18:1(d7) [47.3]; lyso-phosphatidylethanolamine (LPE) 18:1(d7) [10.3]; ceramide (CER) 18:1(d7) [532.2]; monoglyceride (MG) 18:1(d7) [5.5]; diglyceride (DG) 15:0_18:1(d7) [17.0]; triglyceride (TG) 15:0_18:1(d7)_15:0 [67.8]; sphingomyelin (SM) 18:1;2O/18:1(d9) [40.7]; and cholesterol(d7) [254.2]. All the other chemicals were of analytical grade. 

### 2.2. Isolation of NVs from Olive Vegetation Water

OVW was obtained from multicultivar olives, hand collected in the 2021 crop year. The drupes were processed by the two phases oil extraction system of the Bartolomei Olive Oil Mill “Frantoio e Museo dell’Olio Bartolomei”, Montecchio, Terni, Italy. OVW samples were collected in 50 mL tubes and were used fresh or stored at −80 °C until their use for NV isolation. 

The isolation of NVs was carried out by differential centrifugation, followed by density gradient separation [12]. OVW (50 mL) was diluted (1:2, *v*/*v*) in phosphate-buffered saline (PBS) and first centrifuged at 1200× *g* for 20 min, then 3 times at 3000× *g* for 20 min, and finally at 10,000× *g* for 60 min. All centrifugation steps were carried out at 4 °C. The recovered supernatant was filtered with a 0.45 µm pore-sized filter and ultracentrifuged with a Type 70.1 Ti rotor (Beckman Coulture, Indianapolis, IN, USA), at 100,000× *g* for 90 min, at 4 °C, then the pellet was resuspended in 10 mM Tris-HCl (pH 8.6) and submitted to density-gradient ultracentrifugation using a 60% (*w*/*v*) iodixanol working solution (Optiprep, Sigma-Aldrich, Burlington, MA, USA) diluted in 10 mM Tris-HCl (pH 8.6). NV samples were layered on a discontinuous gradient (1.5 mL of 50%, 30% and 10% *w*/*v* gradient cushions) [12]. Ultracentrifugation was carried out with MLS 50 rotor (Beckman Coulter, Indianapolis, Indiana USA), at 100,000× *g* for 18 h, at 4 °C. Ten fractions (500 μL) were collected, diluted 6 times with PBS, then each fraction was pelleted at 100,000× *g* for 90 min, at 4 °C. Pellets were resuspended in PBS and used for the determination of protein content. After ultracentrifugation at 100,000× *g*, detectable amounts of precipitated proteins were present in fraction 5. The presence of NVs in pellet derived from fraction 5 was confirmed by scanning electron microscopy (SEM) and by cryo-transmission electron microscopy (cryo-TEM) (see below). Then, OVW-NVs were used for further determination (number, size distribution, and biochemical composition).

### 2.3. Biophysical Characterization of OVW-NVs

#### 2.3.1. Scanning Electron Microscopy Analysis

SEM analysis was carried out as previously reported [13]. Briefly, OVW-NVs (2 μg) were fixed in glutaraldehyde (2.5%), washed twice with water using Vivaspin (300,000 Da cut-off), and dried on glass coverslips. SEM images were recorded with LEO 1525 scanning electron microscope (Zeiss, Thornwood, NY, USA) equipped with a Gemini column.

#### 2.3.2. Cryo-Transmission Electron Microscopy Analysis

Cryo-TEM analysis was carried out according to previous studies [14]. Briefly, OVW-NVs (5 μL) were placed on a Quantifoil Multi A holey carbon-coated copper R2/1 grid (Quantifoil Micro Tools GmbH, Großlöbichau, Jena, Germany ). Sample vitrification was obtained by blotting excess fluid from the grid and freezing the sample in liquid ethane using a FEI Vitrobot Mark IV plunge freezer. Samples were stored in liquid nitrogen and images were recorded by a Philips CM200FEG microscope operating at 200 kV, equipped with a TVIPS TemCam-F224HD CCD camera and a Gatan 626 Cryo-Holder [14].

#### 2.3.3. Nanoparticle Tracking Analysis

The number and the size distribution of OVW-NVs were evaluated using a NanoSight Model NS300 device (Malvern Instruments, NanoSight Ltd., Salisbury, MD, USA). Five videos of 60 s duration were taken for each determination.

### 2.4. Lipidomic Analysis

#### 2.4.1. Lipid Extraction

NV experimental replicates (5 preparations) were resuspended in 50 µL of PBS for further lipid extraction, which was performed as described by Pellegrino and coworkers [15], with minor modifications. The MMC extraction mixture (9 mL) was prepared by adding 1 mL of IS diluted 1:10 in MeOH, to 2 mL of MeOH, 3 mL of MTBE, and 3 mL of Chlo and 1 mL of the MMC mixture were added to each sample. After vortexing and mixing, samples were centrifuged (16,000× *g* × 10 min, 4 °C), and supernatants recovered and dried using a stream of nitrogen. Lipid extracts were resuspended in a mixture of MeOH-Tol 9:1 *v*/*v*. 

#### 2.4.2. LC-ESI-Q-TOF-MS/MS Analysis

UHPLC-Q-TOF sample analysis was carried out using an Agilent platform consisting of a 1260 Infinity II liquid chromatographer linked through a JetStream source to an Accurate-Mass Q-TOF analyzer. Lipids were resolved on a C18 Acquity UPLC column (100 × 2.1 mm ID, 1.7 μm particles, 100 Å pore size) (Waters Corporation, Milford, MA, USA), operating at flow rate of 0.2 mL/min, at 60 °C. A gradient of water (solvent A), ACN (solvent B), MeOH (solvent C), and IPA (solvent D) was used as mobile phase. Ammonium fluoride (0.2 mM) was added to all solvents; water, MeOH, and IPA were also added with 10 mM ammonium acetate. The gradient was the following: 0–1 min an isocratic gradient at 27% A, 14% B, 24% C, and 35% D; 1–3.5 min a linear gradient at 12.6% A, 17.2% B, 27.2% C, and 43% D; 3.5–10 min a linear gradient at 0% A, 20% B, 30% C, and 50% D; 11–17 min a linear gradient at 27% A, 14% B, 24% C, and 35% D. At 19 min, the run was stopped. The JetStream source ran on the following protocol: 200 °C for the N_2_ gas, 10 L/min for the drying gas, 50 psi for the nebulizer, and 300 °C at 12 L/min for the sheath gas. Spectrometric data were gathered in full scan mode for both positive and negative polarity in the *m*/*z* range 40–1700. To collect as many MS/MS spectra as possible, the pool sample was analyzed five times in an iterative data dependent acquisition (DDA) mode.

#### 2.4.3. Raw Data Processing

Peak-picking, peak alignment, peak annotation, and polarity amalgamation of the raw data was performed with the open-source program MS-DIAL (v. 4.48) [16]. Lipid annotation at the molecular species level was carried out using MS and MS/MS data accordingly with the Lipid Standard Initiative’s (LSI) recommendations [17] (Table S1 in Supplementary Materials). Lipid semi-quantification was performed using the deuterated internal standard Splash Lipidomix for each lipid class at level 2 and 3 of LSI recommendations (https://lipidomicstandards.org/lipid-species-quantification/) (accessed on 15 February 2023).

### 2.5. Metabolomic Analysis

#### 2.5.1. Polar Metabolite Extraction

Polar metabolites were extracted using the method described by Cajka et al. [18], with minor modifications. In brief, OVW and OVW-NVs samples (100 µL) were mixed with a cold MeOH/MTBE mixture (1:5, *v*/*v*). Samples were shaken for 30 s, then 1 vol of 10% *v*/*v* MeOH was added, and samples were shaken again for 30 s and centrifuged (16.000× *g* × 10 min, 4 °C). The bottom aqueous phase (200 µL) was collected, evaporated and resuspended in 100 µL of ACN/H_2_O (4:1, *v*/*v*). Extracts were immediately transferred to an autosampler vial and analyzed without further treatment.

#### 2.5.2. Untargeted LC-ESI-Q-TOF-MS/MS Analysis

UHPLC-Q-TOF analysis of samples was carried out using an Agilent platform consisting of a 1260 Infinity II liquid chromatographer linked through a JetStream source to an Accurate-Mass Q-TOF analyzer. Metabolites were resolved on an Xbridge BHE amide RP-Amide column (HILIC, 150 mm × 2.1 mm, 2.5 μm) (Waters Corporation). The mobile phase consisted of 0.2% aqueous solution of formic acid (solvent A) and 0.2% solution of formic acid in ACN (solvent B). Mobile phase was delivered at a flow rate of 0.35 mL/min under the following gradient procedure: 0–3 min, 90% B; 3–13 min, 48% B; 13–15 min, 48% B; 15–16 min, 90% B; 16–20 min, 90% B. At 22 min, the run was stopped. The column temperature was set at 45 °C, and the injection volume was 10 μL. The ion source operated in both polarities as follows: ion spray 3500 V; gas temperature and sheath gas temperature were set at 280 and 300 °C, respectively; nebulizer (N_2_) 50 psi; and sheath gas flow 12 L/min. DDA was used in the mass *m*/*z* range 40–3200 for both MS and MS/MS, with a collision energy of 30 V.

#### 2.5.3. Raw Data Processing

Peak-picking, peak alignment, peak annotation, and raw data polarity amalgamation were performed with the open-source program MS-DIAL (v. 4.48) [16]. Annotation based on MS and MS/MS data was performed using the NIST 2020 tandem mass library. All metabolites with a total score greater than 60% were considered. Two datasets, one for each polarity, were obtained and then merged in a single table (Table S2 in Supporting Information). At the end of the analytical workflow, a data matrix reporting the relative abundances expressed as the area of each annotated peak in each sample was obtained. This data matrix was used for statistical analysis. 

### 2.6. Proteomic Analysis 

#### 2.6.1. Protein Extraction and Sample Preparation

NV preparations (100 µg) were sonicated in an ultrasonic bath (20 min, on ice), mixed with MeOH/Chlo/water (4/3/1, *v*/*v*/*v*) and centrifuged at 12,000× *g* for 5 min, at 4 °C. The water–organic solvent interface, containing proteins, was mixed with methanol (4 vol) and centrifuged at 14,000× *g* for 5 min, at 4 °C. Protein pellets were solved in 6 M urea, 2 M thiourea, 30 mM Tris-HCl, 10 mM dithiothreitol, 0.1% *w*/*v* Triton X100 and centrifuged at 12,000× *g* for 2 min, at 4 °C. Recovered proteins were then reduced with 10 mM tris(2-carboxyethyl)phosphine for 30 min, at 37 °C, alkylated with 55 mM iodoacetamide for 30 min, at 25 °C, in the dark, and finally insolubilized with acetone at −20 °C, overnight. Proteins were regained by centrifugation at 8000× *g* for 10 min at 4 °C, solubilized in 50 mM triethylammonium bicarbonate (TEAB), and finally proteolyzed with trypsin (enzyme:protein 1:50 *w*/*w*) in 100 mM TEAB, at 37 °C, overnight. 

#### 2.6.2. nanoLC-ESI-Q-Orbitrap-MS/MS Analysis

Protein digests were separated with an UltiMate 3000 HPLC RSLCnano system (Thermo Fisher Scientific, Foster City, CA, USA) and structurally characterized with a Q-ExactivePlus spectrometer (Thermo Fisher Scientific), which was directly linked to the chromatographer by means of a Nanoflex electrospray device. Peptides were initially recovered from a desalting step on µZipTipC18 devices (Millipore, Burlington, MA, USA), and then resolved on an Acclaim PepMapTM RSLC C18 column (150 mm length × 75 μm internal diameter, 2 μm particle size, 100 Å pore size) (Thermo-Fisher Scientific), which was eluted with a gradient of solvent B (water/ACN/formic acid 19.92/80/0.08, *v*/*v*/*v*) in solvent A (water/formic acid 99.9/0.1, *v*/*v*). Mobile phase was delivered at a flow rate of 300 nL/min, using the following gradient parameters: 0–3 min, 3% B; 3–68 min, 30% B; 68–88 min, 60% B; 88–89 min, 95% B; 89–99 min, 95% B. The mass spectrometer operated in DDA mode and acquired MS spectra in the *m*/*z* range 375–1500, at a resolution of 70,000. The eight most abundant ions in each MS scan were subjected to MS/MS analysis, using the following experimental parameters: dynamic *m*/*z* range; normalized collision energy 28%; maximum ion target 120 ms; resolution 17,500; automatic gain control target 100,000; dynamic exclusion 20 s.

#### 2.6.3. Raw Data Processing

Raw MS and MS/MS data were analyzed with Proteome Discoverer v. 2.4 software (Thermo-Fisher Scientific) running the Mascot algorithm v. 2.6.1 (Matrix Science, London, UK) and were subjected to database searching against an *Olea europaea* subsp. Europaea protein database (Oe6 database, 79,910 sequences) (www.denovo.cnag.cat/olive_data accessed on 21 October 2022) [19]. Trypsin was specified as the protease with two maximum missed cleavages. Carbamidomethylation (Cys) was chosen as fixed modification; deamidation (Asn and Gln), pyroglutamate formation (Gln and Glu), and oxidation (Met) were chosen as variable modifications. For parent ions, peptide mass tolerance was specified at ±10 ppm; for fragment ions, peptide mass tolerance was specified at ±0.05 Da. Confidently identified protein candidates must have contained at least 2 peptides associated with an individual Mascot score ≥ 30. Results were filtered to a false discovery rate (FDR) value of 1%. 

#### 2.6.4. Gene Ontology Annotation

The correspondence of the identified protein entries from Oe6 database with UniProtKB accessions for *Olea europea* was manually verified. Automatic functional classification of the identified proteins was performed using Mercator4 v5.0 software [20] and integrated with information obtained manually using UniProtKB website (https://www.uniprot.org accessed on 27 October 2022), according to Interpro, Panther and quickGO annotations.

## 3. Results

### 3.1. OVW Contains NVs

**Figure 1:**
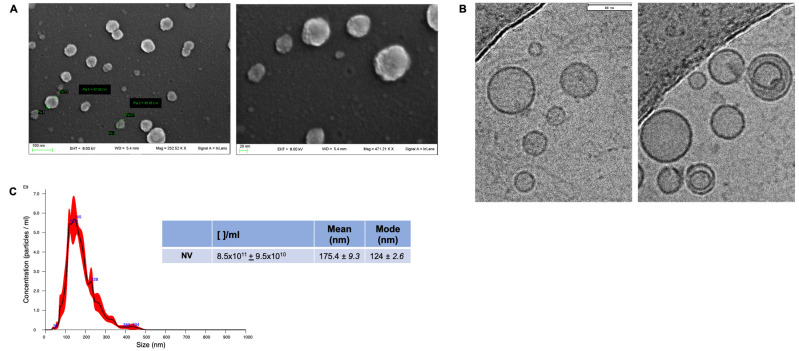
Morphological characterization and size distribution analysis of NVs isolated from OVW. Panels (**A**,**B**) show representative SEM and cryo-TEM images of OVW-NVs, respectively. Panel (**C**) shows representative quantification and size distribution of OVW-NVs. Number of particles/mL ([ ]/mL), mean (nm), and mode (nm) are reported in the table present in the insert of panel (**C**). Data are expressed as mean ± SD (n = 4).

NVs from OVW were isolated as reported in the experimental section. They were characterized for morphology and size distribution by SEM, cryo-TEM and NTA analyses (Figure 1). SEM images revealed that purified OVW-NVs presented a vesicle-like morphology with a round-shaped appearance (Figure 1A). Cryo-TEM images showed that the majority of NVs present in our preparations were spherical nanoparticles delimited by a lipid bilayer; only a few numbers occurred as double vesicles (Figure 1B). The number of vesicles (expressed as protein content) obtained from 50 mL of OVW was 29.45 ± 4.4 µg of proteins, whereas the particle number measured by NTA was 8.5 × 10^11^ ± 9.5 × 10^10^. The NV yields, expressed as the ratio of NV number to protein, were consistent with the range 10^10^ particles/µg of proteins. This value was higher than that reported for mammalian cell-derived extracellular vesicles (EVs), which typically is in the range of 10^9^ [2]. A higher particle–protein ratio has also been reported for NVs isolated from tomato juice vesicles (range of 10^11^–10^12^) [2], thus indicating that vesicles from edible vegetables and fruits contain less protein per vesicle than mammalian cell-derived EVs. Furthermore, NTA analysis showed that most OVW-NVs had an average size of 177.4 ± 9.3 nm (with a mode size 124 ± 2.6 nm) (Figure 1C). 

These results demonstrated the presence of NVs in OVW that were featured by a round-shaped morphology and a size distribution similar to those of vesicles released by mammalian or plant cells.

### 3.2. Lipid Composition of NVs Isolated from OVW

**Figure 2:**
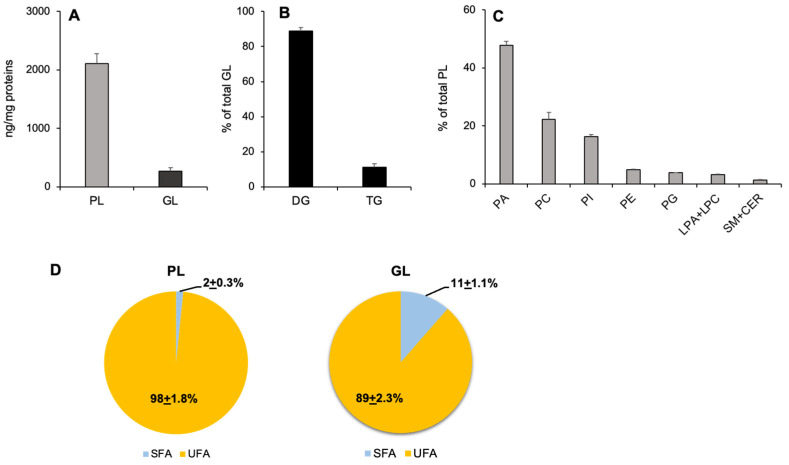
Lipid composition of NVs isolated from OVW. (**A**) The graph shows the phospholipid (PL) and glycerolipid (GL) amount relative to protein content. Data are expressed as mean ± SD (n = 5). (**B**) The graph shows the distribution of detected GL classes. The diglyceride (DG) and triglyceride (TG) amount is expressed as percentage of the sum of all identified GL. Data are expressed as mean ± SD (n = 5). (**C**) The graph shows the PL class distribution. The amount of each PL class is expressed as percentage of the sum of all identified PL species. Data are expressed as mean ± SD (n = 5). (**D**) Pie charts show the percentage of lipids containing only saturated fatty acids (SFA) and lipids containing at least one unsaturated fatty acid (UFA).

UHPLC-Q-TOF analysis of lipids from OVW-NVs provided a dataset of 231 annotated molecular species belonging to 11 lipid classes (Table S1). The quantitative analysis of total annotated lipids revealed that OVM-NVs presented a higher content of phospholipids (PL) per µg of protein, compared to glycerolipids (GL) (Figure 2A). Regarding GL classes, DG represented about 90% of the total GLs (Figure 2B). Among PLs, the most abundant lipid subclasses were phosphatic acid (PA), followed by phosphatidylcholine (PC), phosphatidylinositol (PI), phosphatidylethanolamine (PE), and phosphatidylglycerol (PG) (Figure 2C). Sphyngolipids (sphingomyelin plus ceramide) represented the 1.3 ± 0.1% of total PLs, whereas the lyso-PL (LPA plus LPC) accounted for 3.1 ± 0.3% (Figure 2C). We also evaluated the degree of unsaturation of acyl chain composition calculating the percentage of PL and GL species containing only saturated fatty acids (SFA) or containing at least one unsaturated fatty acid (UFA). Figure 2D clearly shows that OVW-NVs had high levels of lipid molecular species containing UFA, which are precursors of bioactive lipids. Based on these results, OVW-NVs might represent a natural source of bioavailable UFA and their isolation allows the recovery of these valuable lipids from OVW. 

### 3.3. Metabolomic Profile of OVW and OVW-NVs

Untargeted analysis of polar metabolites extracted from OVW and OVW-NVs was carried out by UHPLC-Q-TOF measurements. Metabolites were annotated by spectral matching using the NIST-2020 reference library (Table S2). According to previous studies [11], the metabolome of OVW was found to consist of carbohydrates [oligosaccharides, monosaccharides and their oxidized (carboxylic acid) or reduced (polyalcohol) derivatives], amino acids, phospholipids, and phenolic compounds (Table S2). These classes of metabolites were also detected in OVW-NVs (Table S2). Table 1 shows the 20 most represented annotated metabolites (ordered by peak areas) of OVW and OVW-NVs. In OVW, most of the metabolites belong to the class of carbohydrates and their derivatives. Phenolic compounds, such as 4-O-caffeoylquinic acid and quinic acid, were also present (Table 1). Similarly, the top-ranking metabolites of OVW-NVs were represented by carbohydrates and their derivatives as well as phenolic compounds (4-O-caffeoylquinic, 4,5-dicaffeoylquinic acid, and 5-2’,3’-trimethoxylflavanone). In OVW-NVs, amino acid derivatives and citric acid intermediates were also present in the top-ranking list (Table 1). 

### 3.4. Proteomic Profile of NVs Isolated from OVW

The protein cargo of OVW-NVs was investigated by proteomic procedures based on nanoLC-ESI-Q-Orbitrap-MS/MS. Gene ontology was performed manually according to Interpro, Panther, and quickGo terms; only vicilin-like antimicrobial peptides 2-2 contained an orthologous protein. The identified proteins and their GO annotations are reported in Table 2. Proteomic identification data are detailed in Table S3. Overall, 19 different protein species were detected; among them, four proteins have a nutrient reservoir role (legumin A-like 11S globulin seed storage 2-like, 11S globulin subunit beta-like, and vicilin-like antimicrobial peptides 2-2), two proteins are involved in lipid metabolism (PI-PLC X domain-containing At5g67130-like isoform X2 and glycerophosphodiester phosphodiesterase GDPDL3-like), and one component is linked to carbohydrate metabolism (acidic endochitinase-like). Interestingly, proteins with oxidoreductase/electron transfer activity, including berberine bridge enzyme-like 18, monocopper oxidase SKU5, blue copper-like, and cytochrome b561 and DOMON domain-containing At5g47530-like were also detected. Vicilin-like antimicrobial peptides 2-2 and pathogenesis-related R major form are both involved in defense response. Other proteins detected in this study, namely histone H4-like, actin, and heat shock 70 kDa, were already detected in mammal EVs, as verified by a manual search in the Vesiclepedia database (microvesicles.org accessed on 27 March 2023). Notably, 8 out of 19 proteins identified in OVW-NVs (i.e., actin, heat shock 70 kDa, elongation factor Tu, monocopper oxidase, vicilin, PI-PLC X domain-containing protein, chitinase, pathogenesis-related R major form, and ras-related RABB1c) were already detected in NVs isolated from edible plants and fruits, such as citrus juice, grape, lemon, watermelon, ginger, tomato and garlic [1,2,8,21,22].

## 4. Discussion

It is well known that olive oil, an important component of the Mediterranean diet, has beneficial effects on human health due to the high content of unsaturated fatty acids (i.e., oleic acid) and bioactive compounds. Pomace and OVW represent important by-products generated in large amounts during olive oil production. A common method of valorizing OVW is its utilization for the extraction of bioactive compounds, such as antioxidant phenolic compounds used in nutraceutical and food industry. To date, no studies have isolated NVs from OVW. Results reported in this study indicate that the isolation of NVs from OVW is possible and it might represent an innovative way to valorize this agricultural by-product. 

NVs were isolated from OVW through differential centrifugation followed by a density-gradient centrifugation. The biophysical characterization of our samples by SEM, cryo-TEM and NTA revealed the presence of membrane-enclosed NVs presenting size distribution and morphology similar to nanoparticles already isolated from edible plants and fruits [1,2,7,8]. Since information on biochemical composition is essential to rationalize the biological effects of NVs on mammalian cells, a complete characterization of OVW-NVs was obtained by integrated lipidomic, metabolomic, and proteomic procedures. The results provided novel data to explain the potential use of these NVs as nutraceutical and therapeutical products.

Regarding the lipid composition of NVs, it is well known that lipid membranes determine important biological functions of these vesicles. Lipid analysis of OVW-NVs by UHPLC-Q-TOF revealed that 10% of total detected lipids are GL, consisting mainly of DG, whereas 90% are PL. This result suggested that there may be limited contamination by lipid aggregates in OVW-NVs. Regarding PL class distribution, OVW-NVs were characterized by a high content of PA and PC. This PL composition is different from that of mammalian EVs [13] but is similar to that of NVs isolated from other edible plants, such as tomato [2], ginger [9], and grape [23]. Accordingly, the high amount of PA seems to represent a common feature of NVs isolated from vegetable sources. In plants, PA is a key intermediate of the synthesis of glycerophospholipids and galactoglycerolipids, which are essential compounds for the formation of plant membranes. Furthermore, PA is also a key signaling lipid that regulates important physiological processes, particularly in plant response to environmental stresses [24]. Due to its structural (reduced size of the head group) and chemical (possibility to carry one or two negative charges depending on the environment) features, PA can act as a pH biosensor; its accumulation in a leaflet of a bilayer can favor concave (negative) curvature of the membrane. In plants, animals, and microorganisms, PA is involved in many cellular functions and plays a pivotal role in signaling by facilitating the recruitment to the membranes of several proteins, such as protein kinases. PA carried by grape-EVs was demonstrated to be implicated in the induction of cell proliferation in colon tissue [23]. The process of PA forming membranes in plant NVs has also emerged, regulating membrane fission and fusion and participating in the internalization process by target cells [25]. In addition, PA also plays a role in maintaining the duration and accumulation of plant-derived NVs in the gut [26].

PC, the other abundant phospholipid present in OVW-NVs, was already reported to be present in the membrane of plant-derived NVs [2,9]. Consistently, PA and PC carried by ginger-NVs play an important role in the migration of these vesicles from the intestine to the liver, as well as in the vesicle uptake by intestinal bacteria [27]. Moreover, PA and PC associated with ginger-NVs modulate gut microbiota, determining changes in the composition and localization of bacteria, as well as affecting host physiology [26]. 

Another interesting aspect is that PL and GL associated with OVW-NVs are mainly formed by UFA, which, once internalized, might be used by target cells to synthesize lipid mediators. UFA-containing PL might influence the membrane rigidity of NVs and this, in turn, might modulate the targeting and the internalization by target cells [13]. In this context, the recovery of UFA through the isolation of NVs, as reported in this study, can allow to rescue these valuable compounds, which otherwise would be lost during the extraction of other bioactive molecules from OVW by using organic solvents.

Similar to what was reported in the scientific literature [11], qualitative metabolomic analysis revealed that the main components of OVW are carbohydrates, organic acids, phenolic compounds, and amino acids (Table S1). The same classes of molecules were demonstrated here to be also present in OVW-NVs. Notably, phenolic compounds present in OVW-NVs included phenolic acid derivatives (clorogenic acid, vanillic, cinnamic, and sinapic derivatives) and flavonoids (5,2’,3’-trimethoxyflavanone and tiliroside) (Table S1). Among phenolic acids, clorogenic acid derivatives (4-O-caffeoylquinic acid and 4,5-di-caffeoylquinic acid) were present in the top-ranking metabolites reported in Table 1. In plants, these molecules have a defensive role against biotic or abiotic stresses [28]. Furthermore, clorogenic acid derivatives display many bioactivities, i.e., antioxidant, antibacterial, anticancer, and neuroprotective effects [28]. Among flavonoids, 5,2’,3’-trimethoxyflavanone was present in the top 20 metabolites reported in Table 1. Flavonoids are a class of metabolite present in many fruits and vegetables, which exhibit numerous biological and pharmacological effects [29]. In particular, methylated flavonoids have a higher metabolic stability and oral bioavailability, compared with their unmethylated analogs; this, in turn, enhances their biological effects [30]. Among the top-ranking metabolites carried by OVW-NVs, the presence of 3-dehydroskimic acid, an intermediate of the shikimic acid pathway, is also worth mentioning. The shikimic acid pathway is responsible for the synthesis of many essential metabolites, such as aromatic amino acids, vitamins E and K, ubiquinone, and phenolic compounds. Interestingly, the shikimic acid and its pathway intermediates have attracted significant interest because of their anti-inflammatory and antioxidant activities [31,32].

Proteomic analysis of OVW-NVs evidenced 19 protein components, most of which were already described in vesicles isolated from edible plants and fruits. These proteins are mainly involved in plant stress response and redox systems [1,2,3,7,8,33], or were already described in animal EVs and linked to vesicle functional properties; the latter group included actin, ras-related RABB1c, heat shock 70 kDa, and histone H4-like. 

Growing evidence has confirmed that plant NVs could be considered effective carriers of exogenous proteins into human cells and organs [34]. In this context, it is worth mentioning the detection of heat shock 70 kDa in OVW-NVs, a cytoprotective protein already identified in mammalian EVs as well as in plant- and fruit-derived NVs. Indeed, it has been demonstrated that plant NVs enriched in exogenous heat shock 70 kDa show potential therapeutic benefits for human cells, as this protein fully maintains its specific activity in the vesicle environment and can be delivered to target cells [34,35]. The presence of histone H4-like in OVW-NVs can be viewed in the same perspective, as it has been reported that histones can assist the vesicle endocytotic uptake by target cells [36]. Similar hypotheses can be proposed regarding the presence of actin and ras-related RABB1c in OVW-NVs, as cytoskeletal proteins and ras-associated binding proteins were already reported as being involved in the endo- and exocytic trafficking of EVs [37].

The presence in OVW-NVs of proteins and enzymes linked to cellular redox systems, such as berberine bridge enzyme-like 18, cytochrome b561, and DOMON domain-containing At5g47530, suggests that these vesicles may possibly have antioxidant properties other than those associated with their metabolite content. For example, berberine bridge enzyme-like 18, which belongs to a family of the berberine bridge enzyme-like FAD-dependent oxidases, catalyzes the oxidation of oligosaccharides and acts as a scavenger of different radical cation species, with implications in defense response and redox homeostasis [38]. The cytochrome b561 and DOMON domain-containing protein, which belongs to a family of enzymes widely represented in plant and animal tissues, has a high redox potential, ascorbate reducibility, and capability to transport electrons across biomembranes [39] can be viewed in the same functional perspective. Two other proteins with electron transfer activity identified in OVW-NVs, namely blu copper-like and other mono-copper oxidase-like protein SKU5, can be considered in the same way. These proteins, also known as cupreroxins/phytocyanins, have a large influence on plant growth and resistance. In particular, mono copper oxidase-like protein SKU5 was already identified in small EVs from plant roots with antifungal activity and was associated with a plant defense role [33], analogous to pathogenesis-related proteins and acidic endochitinases also identified here in OVW-NVs. In this context, pathogenesis-related proteins are protease-resistant proteins with a high degree of pathogen specificity [40], while chitinases exert their antifungal and insecticidal activity by hydrolyzing the β-1,4 glysosidic bond in the chitin polymer of the fungal cell wall and insect exoskeleton, producing chito-oligosaccharides and N-acetyl-d-glucosamine. Due to these activities, chitinases are used in food preservation as well as in therapeutic preparations [41]. Notably, chito-oligosaccharides are considered a potential pre-biotic and were also reported as having antimicrobial, anti-inflammatory, and immunostimulant properties [41]. Other proteins identified in OVW-NVs, which were already reported as having antibacterial and antioxidant activity, include 11S globulin seed storage 2-like, 11S globulin subunit beta-like, and vicilin-like antimicrobial peptides 2-2. They are storage components and represent the most abundant proteins in extra virgin olive oil [42]. Finally, this study demonstrated that OVW-NVs contain elongation factor Tu, a conserved GTP-binding protein that, in addition to assisting protein biosynthesis, aids the organism response to various abiotic stresses through its chaperone activity [43]. This protein was already identified in NVs from various edible vegetables and fruits [1,2,7,8,33]. 

## 5. Conclusions and Perspectives

The results of the present study demonstrate the successful isolation of NVs from OVW, a by-product generated by olive oil production, allowing the possibility of an alternative use of this waste according to a circular economy perspective and favoring its valorization through the isolation of vesicles which are natural carriers of bioactive compounds. Furthermore, the procedure used in this study to isolate NVs from OVW is not expensive in terms of starting material and is eco-sustainable (i.e., it does not require the use of organic solvents). 

Biophysical data and information on lipid, metabolite, and protein composition of OVW-NVs demonstrated that the features of these vesicles overlap well with those of bioactive NVs isolated from other edible plants and fruits. The nature of the (macro)molecules identified in OVW-NVs suggests promising beneficial effects that these vesicles could exert on mammalian cells, as some of the cargo components identified here have known antioxidant and anti-inflammatory properties. Moreover, the features of NVs suggest that the above-reported bioactive molecules can reach target cells protected from degradation by exogenous enzymes. In order to demonstrate the potential of OVW-NVs as nutraceutical tools and determine whether NVs isolated from agricultural waste can be alternatives to those obtained from raw plant food, the vesicles’ bioactivity in mammalian cells needs to be explored, assaying the cytotoxicity, antioxidant and anti-inflammatory effects. In addition to this, it is necessary to evaluate the stability in biological fluids and the resistance of OVW-NVs in conditions mimicking the acidic gastric environment. New in-depth studies will evaluate the structural characteristics and functional properties of natural and/or enriched OVW-NVs, with the aim to definitively support their use in nutraceutical and food industries. 

## Figures and Tables

**Table 1:** List of the most represented (20 in number) metabolites identified in OVW and OVW-NVs. Compounds were ordered according to their measured peak area value (mean; n = 4).

OVW		OVW-NVs	
Name	Peak Area	Name	Peak Area
Galactonic acid	4.2 × 10^8^	3 alpha,6 alpha-mannotriose	2.2 × 10^8^
Citric acid	2.0 × 10^8^	3 alpha-galactobiose	1.9 × 10^7^
L-iditol	1.2 × 10^8^	3-Dehydroshikimic acid	1.1 × 10^7^
4-O-caffeoylquinic acid	1.1 × 10^8^	5-(Galactosylhydroxy)-L-lysine	4.0 × 10^6^
D-arabinonic acid	8.3 × 10^7^	4-O-caffeoylquinic acid	2.4 × 10^6^
D-fructose	6.5 × 10^7^	4 alpha-mannobiose	2.1 × 10^6^
2-Keto-L-gulonic acid	3.9 × 10^7^	5,2’,3’-Trimethoxyflavanone	1.6 × 10^6^
D-saccharic acid	2.7 × 10^7^	4,5-Dicaffeoylquinic acid	1.6 × 10^6^
L-fucose	2.4 × 10^7^	L-iditol	1.4 × 10^6^
(-)-Quinic acid	2.3 × 10^7^	Arctiin	1.3 × 10^6^
2-Isopropylmalic acid	1.9 × 10^7^	D-(+)-raffinose	1.2 × 10^6^
Maltitol	1.5 × 10^7^	9-(2,3-Dihydroxypropoxy)-9-oxononanoic acid	1.2 × 10^6^
Ethylvanillin	1.2 × 10^7^	4-Pyridinecarboxylic acid	1.2 × 10^6^
1-O-octyl-2-O-(N-methylcarbamoyl)-sn-glyceryl-3-phosphorylcholine	1.1 × 10^7^	Citric acid	8.3 × 10^5^
Salidroside	1.0 × 10^7^	Choline cation	7.8 × 10^5^
D-psicose	9.6 × 10^6^	Guanosine	6.6 × 10^5^
1,2-Dipalmitoyl-sn-glycero-3-phospho-(1’-myo-inositol)	9.3 × 10^6^	Digalacturonic acid	6.6 × 10^5^
D-mannitol	9.1 × 10^6^	Blood group H disaccharide	6.0 × 10^5^
Guanidinosuccinic acid	8.9 × 10^6^	5-Keto-D-gluconic acid	5.9 × 10^5^
Galactinol	8.7 × 10^6^	Argininosuccinic acid	5.3 × 10^5^

**Table 2:** Protein cargo of OVW-NVs identified by dedicated proteomic analysis using the Oe6 database. Protein name, Oe6 and UniProtKB accession numbers, and GO annotation are reported.

Accession	Description/Protein Name	UniProtKB Accession	GO Annotation
OE6A083970P1	histone H4-like	A0A8S0ST76_OLEEU	M.F.: protein heterodimerization activity (GO:0046982), DNA binding (GO:0003677), structural constituent of chromatin (GO:0030527);
OE6A022576P2	actin	A0A8S0RHH2_OLEEU	C.C.: Cytoskeleton (GO:0005856);
OE6A065156P1	berberine bridge enzyme-like 18	A0A8S0QHL9_OLEEU	C.C.: membrane (GO:0016020);
OE6A060228P2	monocopper oxidase SKU5	A0A8S0QH59_OLEEU	M.F.: copper ion binding (GO:0005507); oxidoreductase activity (GO:0016491).
OE6A010255P1	acidic endochitinase-like	A0A8S0U4B5_OLEEU	M.F.: hydrolase activity, hydrolyzing O-glycosyl compounds (GO:0004553); B.P.: carbohydrate metabolic process (GO:0005975).
OE6A008564P1	elongation factor Tu, mitochondrial	A0A8S0VA50_OLEEU	M.F.: GTPase activity (GO:0003924), GTP binding (GO:0005525), translation elongation factor activity (GO:0003746);
OE6A104529P1	heat shock 70 kDa	A0A8S0QHF9_OLEEU	M.F.: ATP binding (GO:0005524), ATP-dependent protein folding chaperone (GO:0140662); B.P.: protein folding (GO:0006457), response to stress (GO:0006950), response to unfolded protein (GO:0006986).
OE6A042009P1	legumin A-like	A0A8S0UP59_OLEEU	M.F.: nutrient reservoir activity (GO:0045735).
OE6A083307P1	11S globulin seed stor-age 2-like	A0A8S0TZU5_OLEEU	M.F.: nutrient reservoir activity (GO:0045735).
OE6A085162P2	11S globulin subunit beta-like	A0A8S0R9M8_OLEEU	M.F.: nutrient reservoir activity (GO:0045735).
OE6A025388P1	vicilin-like antimicrobial peptides 2-2	A0A8S0V594_OLEEU	M.F.: nutrient reservoir activity (GO:0045735); B.P.: defence response to fungus (GO:0050832), defence response to bacterium (GO:0042742).
OE6A003540P1	blue copper-like	A0A8S0T0I5_OLEEU	M.F.: electron transfer activity (GO:0009055).
OE6A006034P3	PI-PLC X domain-containing At5g67130-like isoform X2	A0A8S0RGA6_OLEEU	B.P.: lipid metabolic process (GO:0006629).
OE6A017022P1	glycerophosphodiester phosphodiesterase GDPDL3-like	A0A8S0Q674_OLEEU	M.F.: phosphoric diester hydrolase activity (GO:0008081).
OE6A044804P1	pathogenesis-related R major form	A0A8S0QR96_OLEEU	B.P.: response to stress (GO:0006950), defense response (GO:0006952), response to stimulus (GO:0050896).
OE6A048391P1	ras-related RABB1c	A0A8S0T0G2_OLEEU	M.F.: GTPase activity (GO:0003924).
OE6A073718P1	cytochrome b561 and DOMON domain-containing At5g47530-like	A0A8S0QLI6_OLEEU	C.C.: membrane (GO:0016020).
OE6A098060P2	bark storage A	A0A8S0RYT1_OLEEU	B.P.: nucleoside metabolic process (GO:0009116). M.F.: catalytic activity (GO:0003824).
OE6A106414P1	cytochrome b561 and DOMON domain-containing At5g47530-like	A0A8S0UDG1_OLEEU	C.C.: membrane (GO:0016020).

B.P. = Biological Process; M.F. = Molecular Function; C.C. = Cellular Component.

## Data Availability

The original contributions presented in the study are included in the article/Supplementary Materials, further inquiries can be directed to the corresponding author.

## Supplementary Materials

The following supporting information can be downloaded at: https://www.mdpi.com/article/10.3390/foods13060835/s1, Table S1: Lipid Molecular Species annotated in OVW-NVs. The amount of each lipid species is expressed as nmol/mg proteins. Data are reported as mean + SD (n = 5); Table S2: List of metabolites annotated in OVW. Compounds were ordered according to their peak area; Table S3. Identification details of the proteins identified in OVW-NVs. Protein accession and description, sum posterior error probability (PEP) score, sequence coverage (%), number of identified peptides, peptide spectrum matches (PSMs), number of identified unique peptides, number of aminoacids in the protein (AAs), theoretical molecular mass of the protein (MW), theoretical isoelectric point of the protein (pI), Mascot identification score, and protein groups number. The identification details of the identified peptides have been reported for each protein and are accessible by clicking on the “+” sign. The identification details of PMSs have been reported for each peptide and are accessible by clicking on the “+” sign. For the description of the columns in the expanded tables of peptides and PSMs, please refer to the Proteome Discoverer User guide available on the web.

## Abbreviations

NVs, nanovesicles; OVW, Olive Vegetation Water; PC, phosphatidylcholine; PE, phosphatidylethanolamine; PS, phosphatidylserine; PG, phosphatidylglycerol; PI, phosphatidylinositol; PA, phosphatidic acid; LPC, lysophosphatidylcholine; LPE, lysophosphatidylethanolamine; CER, ceramide; MG, monoglyceride; DG, diglyceride; TG, triglyceride; SM, sphingomyelin; PBS, phosphate-buffered saline; SEM, scanning electron microscopy; cryo-TEM, cryo-transmission electron microscopy; NTA, nanoparticles tracking analysis; EVs, extracellular vesicles; GL, glycerolipids; PL, phospholipids; SFA, saturated fatty acids; UFA, unsaturated fatty acids.
